# Supramolecular Nano-Encapsulation of Anabasine Reduced Its Developmental Toxicity in Zebrafish

**DOI:** 10.3389/fchem.2020.00134

**Published:** 2020-02-28

**Authors:** Yan Gao, Xue Yang, Ziyi Wang, Zhangfeng Zhong, Yuanjia Hu, Yitao Wang

**Affiliations:** State Key Laboratory of Quality Research in Chinese Medicine, Institute of Chinese Medical Sciences, University of Macau, Macau, China

**Keywords:** supermolecular, anabasine, cucurbit[7]uril, toxicity, zebrafish

## Abstract

Anabasine (ANA), a major piperidine alkaloid originally isolated from wild tobacco trees (*Nicotiana glauca*), has been known to induce serious developmental toxicities such as skeletal deformities in livestock and humans. In this study, we thoroughly investigated the supramolecular nano-encapsulations of ANA by an artificial nanocontainer, cucurbit[7] uril (CB[7]), and examined the influences of the nano-encapsulation on ANA's inherent developmental toxicities on a zebrafish model. We have shown that CB[7] formed 1:1 host-guest inclusion complexes with ANA via a relatively high binding strength [*K*_*a*_ of (7.45 ± 0.31) × 10^4^ M^−1^] in an aqueous solution, via UV-vis and ^1^H nuclear magnetic resonance spectroscopic titrations, as well as isothermal titration calorimetry titration. As a consequence, CB[7] significantly attenuated the developmental toxicity of ANA on zebrafish *in vivo*. In contrast, for a comparative purpose, β-CD didn't exert any influence on the toxicity of ANA due to its weak binding with ANA, which was not even measurable via either spectroscopic methods or ITC titration. This is the first head-to-head comparison of this pair of nanocontainers, CB[7] and β-CD, on their potential roles in influencing the toxicity of guest molecules and the results suggested that CB[7] could become a more promising functional excipient for reducing the inherent toxicities of active pharmaceutical ingredients, particularly alkaloids that may form relatively strong host-guest binding species with the host.

## Introduction

**Figure 1:**
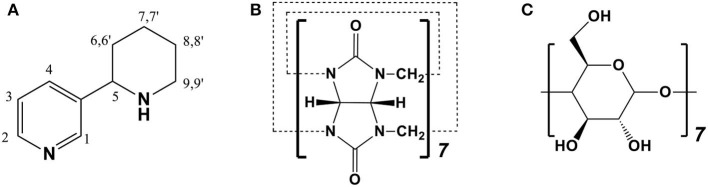
Chemical structures of **(A)** anabasine (ANA), **(B)** cucurbit[7]uril (CB[7]), and **(C)** beta-cyclodextrins (β-CD).

Anabasine (ANA) (Figure 1A), a major piperidine alkaloid originally isolated from wild tobacco trees (*Nicotiana glauca*), has been known to induce serious developmental toxicities, particularly skeletal deformities in livestock (such as goats and swine) and humans (Keeler et al., 1984; Panter et al., 1990; Green et al., 2013a,b). When human beings were presented with poison symptoms after accidental injection of tobacco plant (*N. glauca)*, ANA was identified as a crucial substance causing clinical toxicity (Manoguerra and Freeman, 1982; Mellick et al., 1999; Sims et al., 1999; Mizrachi et al., 2000). ANA is a potential nicotinic acetylcholine receptor (nAChR) agonist, existing in tobacco smoke in a trace amount as it is mainly located from the stalks of cultivated tobacco (Jacob et al., 1999; Daly, 2005; Green et al., 2013b). Thus, ANA could be applied as an indicator of smoking (Jacob et al., 1999; Daly, 2005; Green et al., 2013b).

Cucurbit[*n*]uril (CB[*n*], *n* = 5–8, 10 in particular) is pumpkin-like nano-sized molecule comprised of *n* glycoluril connected by n pairs of methylene bridges (Lagona et al., 2005; Assaf and Nau, 2015). Compared to the well-known cyclodextrins (CDs) family, CB[*n*] family is relatively new group of macrocycles, comparable in many ways to CDs (Shetty et al., 2015). In the CB[*n*] family, CB[7] (Figure 1B) has caught the most attentions from researchers and has displayed a broad range of potential applications due to its promising biocompatibility, appropriate cavity size, superior molecular recognition ability and moderate solubility in water (Uzunova et al., 2010; Oun et al., 2014; Chen et al., 2015b; Kuok et al., 2017; Yin and Wang, 2018; Zhang et al., 2018). Although CB[7] and β-CD (Figure 1C) have rather similar internal cavity volume (279 Å^3^ vs. 263 Å^3^), CB[7] has generally higher binding affinities (*K*_*a*_ = 10^4^-10^17^ M^−1^) with a variety of guest species than those of β-CD (*K*_*a*_ = 10^2^-10^5^ M^−1^).^13^ It has been reported that CB[7] may become an effective pharmaceutical excipient with several benefits (Chen et al., 2015a; Li et al., 2016; Yang et al., 2016, 2017; Huang et al., 2018a; Zhang et al., 2019). For example, it was demonstrated that CB[7] had the promising potential to serve as a taste-masking nanocontainer (Yang et al., 2017). Moreover, Zhang et al. also demonstrated that CB[7] may act as an oral antidote for paraquat poisoning (Zhang et al., 2019).

On the other hand, zebrafish (*Danio rerio*) is a common organism model being used in various biomedical researches. This teleost has high fecundity (200–300 eggs are laid by one adult female fish every week) and rapid *ex utero* development. The early development stages of zebrafish have been well-characterized. As zebrafish's embryo is transparent, the morphologies and internal organs could be well-observed by microscopy without sacrifice (Kimmerl et al., 1995; McGrath and Li, 2008). Thus, zebrafish is an ideal animal model for screening chemical compounds for toxicities. In the present study, we selected natural toxic compound, ANA, as the guest toxic molecule, examined its nano-encapsulation by the synthetic nanocontainer, CB[7], and investigated the possible role of macrocyclic nano-encapsulation in influencing the toxicity of ANA on zebrafish.

## Materials and Methods

### Ethics Statement

The animal experiments and associated experimental protocols were inspected and approved by the Animal Ethics Committee, University of Macau.

### Reagents and Instrumentations

Anabasine (ANA) was purchased from Sigma Aldrich (USA), and β-CD was purchased from J&K (Shanghai, China). These chemicals were used without any further purification. CB[7] was prepared based on a published method by Day et al. (2001). Thermo LTQ Orbitrap XL coupled with an ESI/APcl multiprobe was employed to acquire ESI-MS data. Six hundred megahertz NMR (Bruker) spectrometer was employed to collect NMR spectra. A Confocal Imaging System (Olympus DSU) was used to record morphology of zebrafish.

### Maintenance of Zebrafish

Zebrafish were originally procured from the ZFIN (Oregon). Zebrafish were kept and maintained at the Zebrafish Aquarium in the Institute of Chinese Medical Sciences, University of Macau. Zebrafish were maintained, bred and selected according to the Zebrafish Handbook, as was previously described (Chen et al., 2015b).

### ^1^H NMR Spectroscopy

Solution of ANA (50 μM) was prepared for ^1^H NMR characterization in D_2_O (pD = 7). An deuterated solution of ANA was mixed with different amount of CB[7], 0.6, 1.2, and 2.1 equiv. that of ANA, respectively, without changing the concentration of ANA. These solutions were characterized via ^1^H NMR spectroscopy (NS = 128 times).

### UV-vis Spectroscopy

Two respective aqueous solutions (pH = 7) of ANA (50 μM) and CB[7] (50 μM) were prepared for Job's plot experiment. The samples were prepared by mixing ANA and CB[7] solutions of different volumes to make [CB[7]]/[[ANA] + CB[7]] from 0 to 1.0 at intervals of 0.1. During UV-vis spectroscopic titrations, an aqueous solution of ANA (50 μM) and an aqueous solution of 50 μM ANA and 250 μM CB[7] were prepared (pH = 7). Mixing of the two solutions with different volumes to have [CB[7]]/[ANA] varying from 0 to 5.0 at intervals of 0.5 was conducted and followed by UV-vis absorbance measurements. Same method was employed to prepare β-CD solution for UV-vis spectroscopic titrations with ANA.

### ESI-MS Experiment

Aqueous solutions (pH = 7) containing the ANA (50 μM) and 5 equiv. CB[7] (250 μM), and neutral aqueous solution containing ANA (50 μM) and β-CD (1 mM) were prepared. They were subsequently filtered for ESI-MS analysis.

### Isothermal Titration Calorimetry (ITC) Experiment

Aqueous solutions of ANA (200 μM) and β-CD (4 mM), ANA (100 μM) and CB[7] (1.5 mM) were prepared, respectively. The four solutions were subsequently degassed by centrifuge (13,000 rpm, 10 min) prior to use for titration. 0.2 mL ANA (200 μM) solution was placed in the sample cell, while 0.04 mL β-CD (4 mM) was placed to syringe for injection. Similarly, ANA (0.2 mL, 100 μM) was the sample cell, and 0.04 mL CB[7] was placed to syringe for injection. The ITC titrations were performed by titrating 19 aliquots (2 μL per drop) of β-CD or CB[7] solutions at room temperature, and the heat evolutions were recorded.

### Developmental Toxicity Evaluations of ANA in Zebrafish

Wild type zebrafish embryo was employed as the *in vivo* model to assess the toxicity of ANA in the absence and in the presence of macrocyclic containers. Zebrafish embryos were known to take up chemical species mainly via skin (McGrath and Li, 2008), thus fish were simply incubated in media containing these compounds. The viable hatching rate, survival and morphology of zebrafish embryo were recorded. Zebrafish embryo of 24 hpf were randomly selected and distributed into a 6-well microplate (25–30 embryos/well). To evaluate the developmental toxicity of ANA alone in zebrafish, embryos were incubated in ANA solutions of 4 different concentrations (50, 100, 200, and 300 μM) without CB[7] or β-CD. Of important note, CB[7] was previously investigated to show excellent biocompatibility toward zebrafish embryos in these concentration range (Chen et al., 2015b). Additional experimental groups of embryos were, respectively, incubated in ANA solutions of these 4 different concentrations (50, 100, 200, and 300 μM) with 300 μM CB[7] and 1 mM β-CD (to ensure large excess of β-CD), respectively, for 2 days. At the end of incubation, the number of hatched larvae from each experimental group was recorded and the hatchability rate was analyzed. During the assessment of survival rate, zebrafish embryos (24 hpf) were dechorionated, and the dechorionated embryos were randomly selected and distributed into a 6-well microplate (25–30 embryos/well) and treated with ANA of different concentrations (50, 100, 200, and 300 μM) with or without 300 μM CB[7] or 1 mM β-CD, respectively, for 48 h. Survival rate was recorded by the end of the experiment. In addition, the morphology of zebrafish was measured via an Olympus Confocal Imaging System for further visual assessment.

## Results and Discussions

### Encapsulations of ANA by CB[7] and β-CD

**Figure 2:**
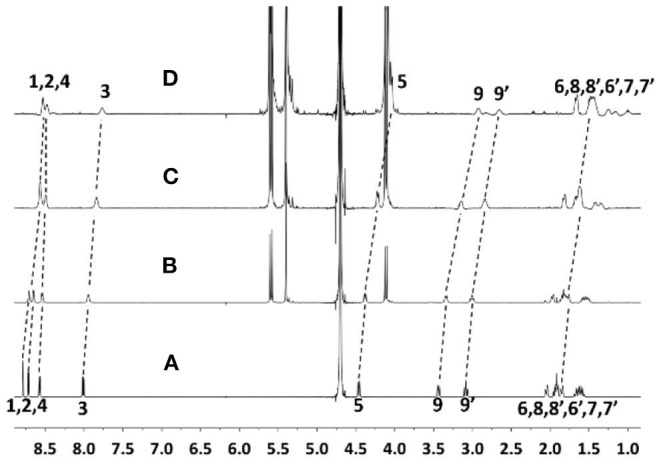
^1^H NMR spectra of ANA (50 μM) **(A)** without and with **(B)** 0.6, **(C)** 1.2, and **(D)** 2.1 equiv. CB[7] in D_2_O (pD = 7) at room temperature.

The supramolecular nano-encapsulation of ANA by CB[7] was investigated via ^1^H NMR and UV-Vis spectroscopic titrations and ITC titration. As shown in Figure 2, all the protons of ANA exhibited an upfield shift after adding escalating concentration of CB[7], suggesting a full encapsulation of majority of ANA into the nano-cavity of CB[7], as the protons were shielded by the non-poplar cavity. Seilkhanov et al. studied the binding behavior between ANA and β-CD by NMR spectroscopy. Based on their results, ANA forms 1:1 host-guest binding pairs with β-CD with the piperidine fragment being included inside the cavity of CD (Seikhanov et al., 2016).

**Figure 3:**
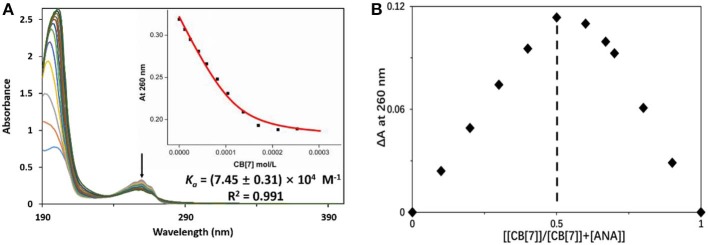
**(A)** UV-vis measurements of ANA (50 μM) in the presence of increasing concentration of CB[7] (up to 250 μM) in aqueous solution (pH = 7). Inset: the fitting curve of the titration data at 260 nm; **(B)** Job's plot of ANA@CB[7] system (with [ANA]+[CB[7]] = 50 μM) based on a continuous variation titration in aqueous solutions (pH = 7).

UV-vis spectroscopic study was employed to assess the complexation ratio and binding affinity (*K*_*a*_) between ANA and CB[7] (Figure 3). The Job's plot of ANA@CB[7] complex (Figure 3A) reached a maximum at 260 nm when [[CB[7]]/[CB[7]] + [ANA]] was 0.5, indicating a 1:1 complexation stoichiometry between ANA and CB[7]. UV-vis spectroscopic titration (Figure 3B) of ANA [0.05 mM) with increasing amounts of CB[7] (from 0 to 0.25 mM)] showed a moderate hypochromic shift. The non-linear least-squares fit was used for calculating the binding constant, affording a *K*_*a*_ of (7.45 ± 0.31) × 10^4^ M^−1^ for ANA@CB[7] pair.

**Figure 4:**
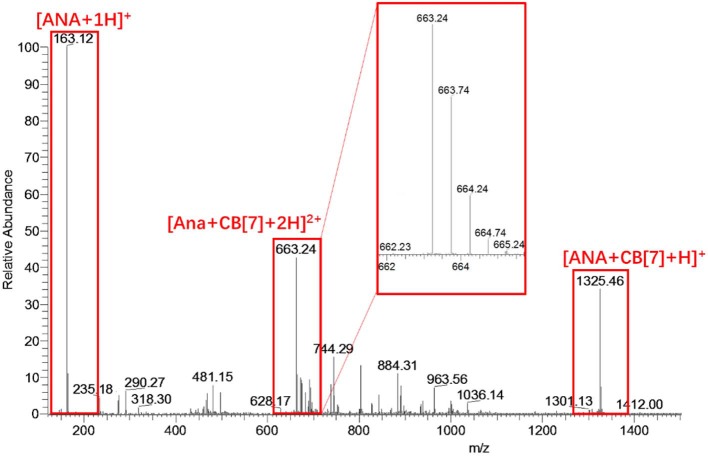
ESI-MS result of an aqueous solution of ANA and CB[7]. The singly-charged peak at *m/z* = 1325.46 and the doubly-charged peak at *m/z* = 663.24 suggested a likely 1:1 complexation ratio of ANA@CB[7].

ESI-MS analysis was conducted to further confirm the complexation ratio between ANA and CB[7]. As shown in Figure 4, a doubly-charged peak at *m/z* = 663.24 and a singly-charged peak at *m/z* = 1325.46 were observed, corresponding to the [ANA+CB[7] + 2H]^2+^ complex (calculated *m/z* = 663.23) and [ANA+CB[7]+H]^1+^ complex (calculated *m/z* = 1325.46), respectively, confirming the 1:1 complex ratio between ANA and CB[7]. For comparative purpose, ESI-MS analysis of the mixture of ANA and β-CD did not generate any peaks attributed to host-guest complexations, implying that β-CD and ANA binds too weakly in aqueous solutions.

**Figure 5:**
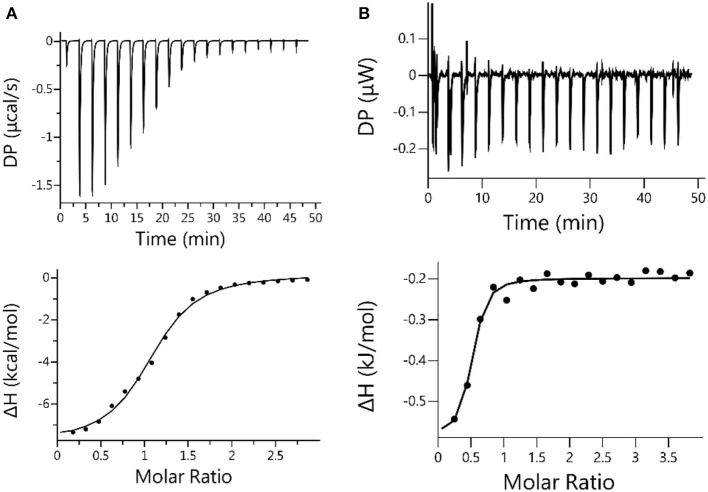
**(A)** Microcalorimetric measurements of ANA when titrated with CB[7] in water at room temperature. Top: Evolved thermogram during the titration. Bottom: Plot of ΔH against the molar ratio between CB[7] and ANA during the titration, with the best fit by using the “one set of binding sites” binding mode; **(B)** Microcalorimetric measurements of ANA when titrated with β-CD in water at room temperature. Top: Evolved thermogram of during the titration. Bottom: Plot of ΔH against the molar ratio between β-CD and ANA, with the best fit by using the “one set of binding sites” binding mode.

ITC titration was conducted to further verify the relative strong binding between CB[7] and ANA. The result generated *K*_*a*_ = 1.40 × 10^5^ M^−1^ shown in Figure 5A, consistent with UV-Vis titration results (Figure 3B). For a comparative purpose, ITC analysis of the mixture of ANA and β-CD was also conducted, however no meaningful thermal evolvement was observed (Figure 5B), suggesting the binding between β-CD and ANA was too weak in aqueous solutions. Collectively, all these results supported that CB[7] formed a relatively stable 1:1 supramolecular complex with ANA with a complexation affinity *K*_*a*_ of (7.45 ± 0.31) × 10^4^ M^−1^, whereas the binding between β-CD and ANA was much weaker.

### The Impact of Supramolecular Nano-Encapsulation on ANA's Inherent Developmental Toxicities in Zebrafish

**Figure 6:**
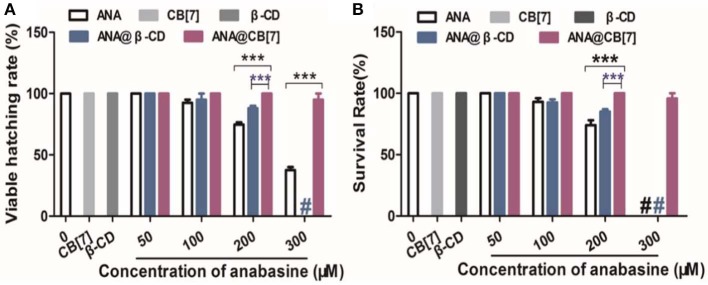
**(A)** The hatching percentage, and **(B)** survival rate of zebrafish embryos treated with ANA w and w/o CB[7] (300 μM) or β-CD (1 mM), respectively, for 2 days. Data are presented as mean ± S.E.M. (*n* = 25–30). *** *denotes P* < 0.001. # denotes that all zebrafish embryos/larva were dead.

Zebrafish embryos were incubated in different concentrations of ANA with or without CB[7] or β-CD. As was observed in Figure 6A, the hatching rate of the fish was decreased with increasing concentrations of ANA, confirming a dose-dependent developmental toxicity of ANA in zebrafish. Embryos incubated with the highest concentration (300 μM) of ANA led to <50% hatching rate. Very interestingly, the hatching rate of embryonic fish incubated with ANA@CB[7] complex exhibited almost the same rate as that of the control group (medium only), suggesting that CB[7] could dramatically alleviate the embryonic developmental toxicity of ANA in fish embryos. Of note, CB[7] alone did not exhibit any signs of toxicity in this study. In contrast, the hatching rate of embryos treated with ANA in the presence of a high concentration of β-CD decreased dramatically with increasing amount of ANA, and the viable hatching rate turned to zero with 300 μM ANA with 1 mM β-CD, which is dramatically lower than that in the free ANA treated group, suggesting that β-CD even somewhat worsened the toxicity of ANA. Similarly, the survival rate of zebrafish treated with free ANA exhibited a dose-dependent manner. In contrast, the survival rate of embryos incubated with ANA@CB[7] complex was maintained the same during 48 h incubation in spite of the increasing doses of ANA. While β-CD showed little effect on the toxicity of ANA in the survival study (Figure 6B).

**Figure 7:**
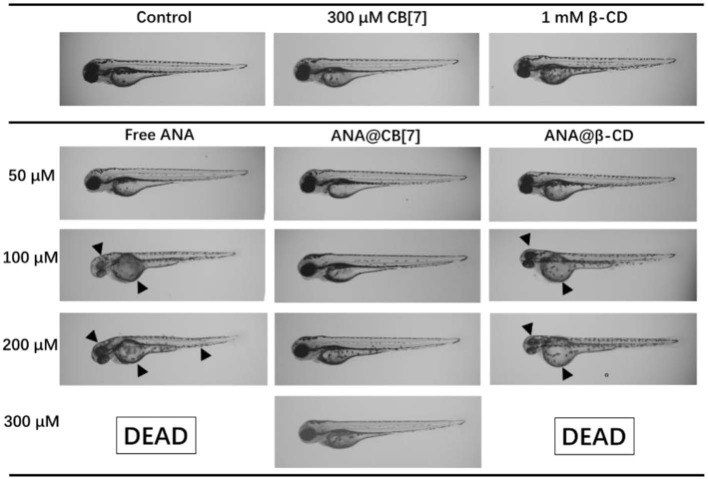
Morphology of representative zebrafish larvae (72 hpf) upon treatment with ANA w and w/o CB[7]/β-CD, respectively, for 48 h. Black arrowheads point to the abnormal morphologies.

In addition, the developmental morphologies of the fish were further examined. As shown in Figure 7, embryo larvae treated with ANA@CB[7] complex displayed normal morphology as well as usual developmental progress, comparable to those in the blank group. In a dramatic contrast, zebrafish larvae treated with free ANA and the mixture of ANA and β-CD both showed obvious developmental malformation, including the malformation of notochord, such as lordosis or cyphosis, yolk deformity, and abnormal head shape. Moreover, with the highest concentration (300 μM) of ANA with and without β-CD, 100% larva were dead. Taken together, CB[7] exhibited significant inhibition effect on the developmental toxicity of ANA whereas β-CD exhibited negligible or even negative effects.

Indeed, several works have previously demonstrated that the CB[*n*]s may alleviate toxicity of guest or included species. For instance, CB[8] played a critical role on formulating a safe, photo-responsive herbicide formulation (Gao et al., 2018). CB[7] was previously reported to inhibit the non-specific teratogenic toxicity of a couple of representative pesticides on a zebrafish model, through a facile supramolecular encapsulation (Yang et al., 2018). In addition, CB[7] was studied for its encapsulation of trazodone (TZ), an anti-depression drug approved by the FDA, but can cause liver damage due to its toxicity (Huang et al., 2018b). CB[7] formed binary host-guest complex with TZ in a relatively strong complexation affinity, and *in vitro* and *in vivo* studies indicated that the hepatotoxicity from TZ were alleviated by CB[7] encapsulation Our present ANA@CB[7] study, as a representative case for guest alkaloid, further supported that CB[7] could become a functional excipient to potentially alleviate undesired toxicities of selected drug or bioactive molecules. And this investigation is the first one to compare the influences of two macrocyclic molecules, CB[7] and β-CD, on the toxicity of a guest species, which may provide new insights on drug's formulations.

## Conclusions

In summary, CB[7] was shown to form a 1:1 stable host-guest complex with a natural alkaloid—ANA, with a relatively high binding affinity. The supramolecular encapsulation exhibited promising inhibitory effects on the developmental toxicity of ANA in zebrafish. In addition, for comparative purpose, β-CD was also investigated in this study, however exhibited negligible effects on the toxicity of ANA. Although β-CD has served as a pharmaceutical excipient for decades, our present study demonstrated that CB[7] might become an important, complementary excipient to β-CD, particularly for reducing undesired toxicity of active compounds such as alkaloids.

## Data Availability Statement

The datasets generated for this study are available on request to the corresponding author.

## Ethics Statement

The animal study was reviewed and approved by the Animal Ethics Committee University of Macau.

## Author Contributions

The project was conceptually designed by YG, YH, and YW. The majority of the experiments were performed by YG and XY, assisted by ZW and ZZ. Data analysis and interpretation were carried out by YG, XY, and YH. The manuscript was prepared by YG, XY, YH, and YW. All authors discussed the results and implications and commented on the manuscript.

### Conflict of Interest

The authors declare that the research was conducted in the absence of any commercial or financial relationships that could be construed as a potential conflict of interest.
